# Indoximod: An Immunometabolic Adjuvant That Empowers T Cell Activity in Cancer

**DOI:** 10.3389/fonc.2018.00370

**Published:** 2018-09-11

**Authors:** Eric Fox, Thomas Oliver, Melissa Rowe, Sunil Thomas, Yousef Zakharia, Paul B. Gilman, Alexander J. Muller, George C. Prendergast

**Affiliations:** ^1^Department of Hematology-Oncology, Lankenau Medical Center, Wynnewood, PA, United States; ^2^Lankenau Institute for Medical Research, Wynnewood, PA, United States; ^3^Holden Comprehensive Cancer Center, University of Iowa, Iowa City, IA, United States; ^4^Sidney Kimmel Cancer Center, Thomas Jefferson University, Philadelphia, PA, United States

**Keywords:** immunometabolism, immune adjuvant, Immunotherapy, immuno-chemotherapy, immuno-radiotherapy

## Abstract

Exploding interest in immunometabolism as a source of new cancer therapeutics has been driven in large part by studies of tryptophan catabolism mediated by IDO/TDO enzymes. A chief focus in the field is IDO1, a pro-inflammatory modifier that is widely overexpressed in cancers where it blunts immunosurveillance and enables neovascularization and metastasis. The simple racemic compound 1-methyl-D,L-tryptophan (1MT) is an extensively used probe of IDO/TDO pathways that exerts a variety of complex inhibitory effects. The L isomer of 1MT is a weak substrate for IDO1 and is ascribed the weak inhibitory activity of the racemate on the enzyme. In contrast, the D isomer neither binds nor inhibits the purified IDO1 enzyme. However, clinical development focused on D-1MT (now termed indoximod) due to preclinical cues of its greater anticancer activity and its distinct mechanisms of action. In contrast to direct enzymatic inhibitors of IDO1, indoximod acts downstream of IDO1 to stimulate mTORC1, a convergent effector signaling molecule for all IDO/TDO enzymes, thus possibly lowering risks of drug resistance by IDO1 bypass. In this review, we survey the unique biological and mechanistic features of indoximod as an IDO/TDO pathway inhibitor, including recent clinical findings of its ability to safely enhance various types of cancer therapy, including chemotherapy, chemo-radiotherapy, vaccines, and immune checkpoint therapy. We also review the potential advantages indoximod offers compared to selective IDO1-specific blockade, which preclinical studies and the clinical study ECHO-301 suggest may be bypassed readily by tumors. Indoximod lies at a leading edge of broad-spectrum immunometabolic agents that may act to improve responses to many anticancer modalities, in a manner analogous to vaccine adjuvants that act to boost immunity in settings of infectious disease.

## Introduction

Immune therapy has risen to the forefront of cancer therapy in recent years, providing a new approach to cancer therapy, and in some instances has begun to shift the paradigm of cancer care from chemotherapy to immunotherapy. One of the factors crucial to the success of immunotherapy is reversing tumor-mediated immunosuppression (1). The tryptophan catabolic enzyme indoleamine 2,3-dioxygenase-1 (IDO1) has received a great deal of attention as a driver of tumor-mediated suppression (2–4). IDO1 has been shown to be active in many human cancers and its expression has been associated widely with poor prognosis (5, 6). Accordingly, inhibitors of the enzymatic activity and effector functions of IDO1 have been developed as tools to leverage cancer therapy (7).

Elevated tryptophan catabolism as a characteristic of patients with cancer was initially reported over 60 years ago (8). The basis for this observation and later observations in various types of cancer patients was not clear until IDO1 was discovered in the 1960s. An association of elevated tryptophan cata olism with inflammation was established in the 1970s−1980s with demonstrations that IDO1 is induced strongly in the lungs by LPS, viral infection and interferon (9–12). In a seminal line of work in the late 1990s by Munn and Mellor and colleagues, tryptophan catabolism was implicated in immunosuppression during pregnancy, based on the preferential sensitivity of T cells to tryptophan deprivation leading to an impairment of antigen-dependent T cell activation (13–15). In these studies, the key probe in defining this mechanism of immune tolerance was the racemic compound 1-methyl-tryptophan (1MT), a tryptophan mimetic with complex IDO inhibitory effects discussed further below. Indeed, much of the huge amount of subsequent work on IDO and disease pathogenesis has relied on this compound, including most importantly cancer studies.

A causal relationship between IDO1 activity and cancer growth was founded by pivotal studies in the 2000s that have been reviewed in detail elsewhere (7). IDO1 was found to be overexpressed widely in human cancers and 1MT could slow the growth of murine tumors (6, 16, 17). IDO1 overexpression in cancer cells was linked genetically to inactivation of BIN1 (18), a tumor suppressor gene widely attenuated in human cancer (19). Loss of BIN1 empowers IFN/STAT and NFkB mediated IDO1 transcription and later studies also implicated the RAS/MAPK, COX2, and PI3K pathways in driving IDO1 expression (18, 20–22). Interestingly, drugs that target molecules relying on these pathways may act in part by indirectly blocking IDO1 expression, such as the case with imatinib (Gleevec) (23). Pharmacological blockade with 1MT or true catalytic inhibitors of IDO1 enzyme were found to display unimpressive efficacy unless combined with DNA damaging therapies, which led to regression of otherwise unstoppable tumors (18, 24, 25). Preclinical genetic proofs of IDO1 as a valid therapeutic target in cancer were enabled in IDO1-deficient mice, where fundamental connections between IDO1 expression and cancerous inflammatory programs were also established (21, 26, 27).

**Figure 1:**
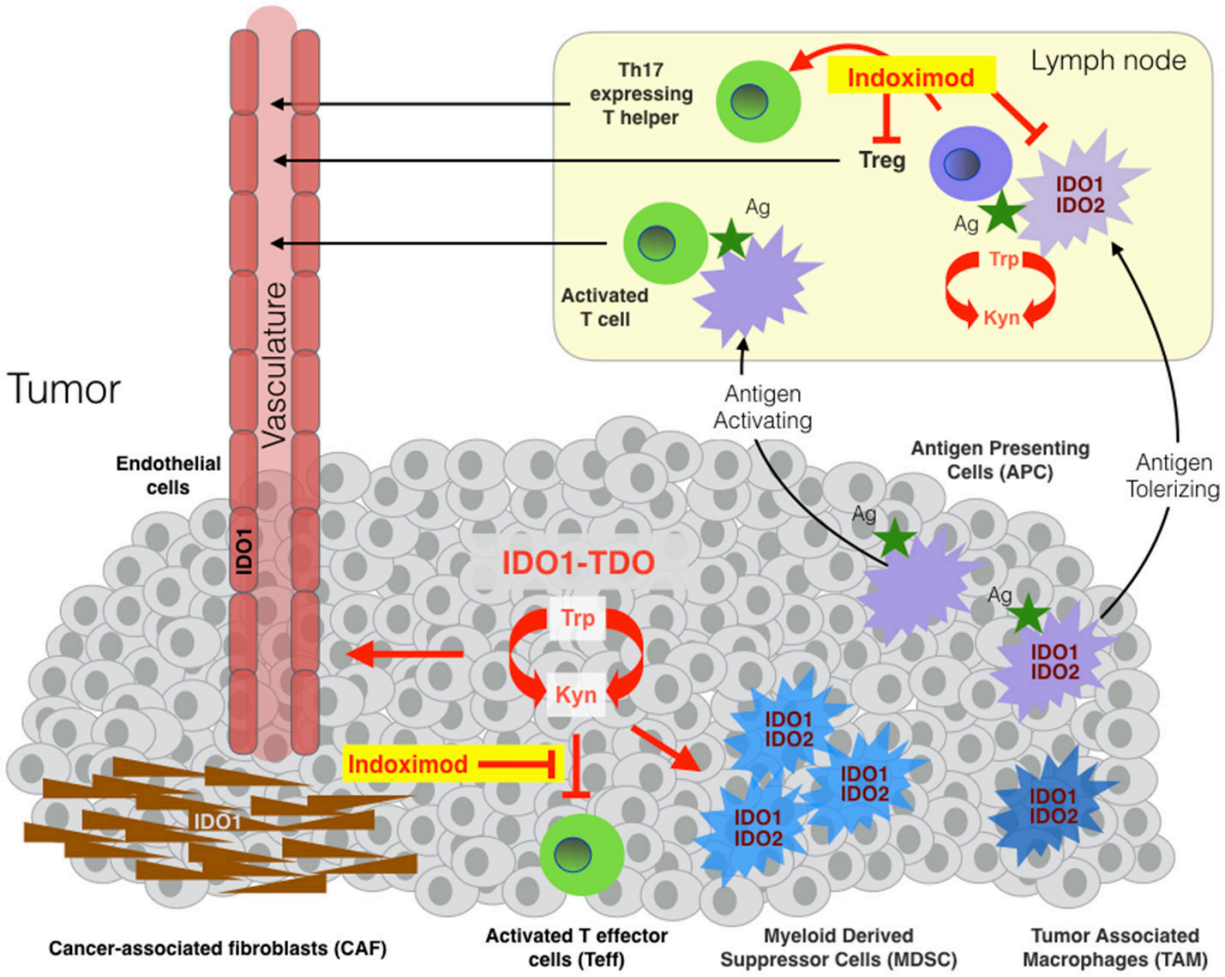
Sites of indoximod action in cancer. IDO1, TDO, and IDO2 are expressed variously in malignant, immune, stromal and vascular cells in the tumor microenvironment and in antigen-presenting cells (APC) of tumor-draining lymph nodes. TDO and IDO2 are relatively more narrowly expressed than IDO1 in human cancers, with TDO overexpressed in some tumors independently or in parallel with IDO1 and IDO2 expressed in antigen-presenting cells including B cells where it may influence IDO1 function (28). Tryptophan catabolism in tumor cells leads to locoregional generation of kynurenine at the cost of tryptophan, enabling suppression of local T effector cells (Teff), licensing and recruitment of myeloid-derived suppressor cells (MDSC) and support of a tumor-enabling vasculature. The tumor microenvironment variously recruits cancer-associated fibroblasts, vascular endothelial and inflammatory myeloid cells that express IDO1 and/or IDO2. Tumor antigens absorbed by IDO1/IDO2-expressing antigen-presenting cells (APC) rove to draining lymph nodes where they promote the formation and activation of T regulatory cells (Tregs). Studies suggest that indoximod enables Teff in tumors and attenuate in draining lymph nodes, in the latter case by acting on dendritic cells leading to suppression and/or reprogramming of Tregs and the formation of Th17-expressing T helper cells.

In the tumor microenvironment or draining lymph nodes, IDO1 activity suppresses the function of T effector cells (Teff) and natural killer (NK) cells and promotes the induction and activation of T regulatory cells (Treg) and the activation, recruitment and expansion of myeloid-derived suppressor cells (MDSC) (Figure 1) [Fallarino(21, 29–36)]. IDO1 effector functions are mediated by the tryptophan catabolite kynurenine (Kyn) and by two stress signals generated by locoregional deprivation of tryptophan (7), as discussed further below. Investigations of IDO1 in immune tolerance have focused heavily on antigen-presenting dendritic cells where IDO1 is upregulated by interferons, TLR ligands and other immune signals (37). Beyond its roles in provoking Treg development, IDO1 also acts in certain dendritic cells to directly suppress effector T cell responses (38, 39).

## Biological roots of indoximod as an immunometabolic adjuvant for cancer therapy

The racemic compound 1-methyl-D,L-tryptophan (1MT) was first described as a competitive inhibitor of the IDO1 enzyme by Cady and Sono in the early 1990s (40). After the seminal demonstration that 1MT could elicit allogeneic conceptus rejection by ablating T cell tolerance to paternal fetal antigens (13), 1MT was shown to weakly retard the growth of cancer cells in mouse tumor graft or spontaneous transgenic models of cancer (16, 17). While the anticancer effects of 1MT were unremarkable as monotherapy, its striking therapeutic power was revealed in combinations with DNA damaging chemotherapy which elicit regressions of otherwise recalcitrant tumors (18). This discovery was an important advance in providing the first indication of how to use an IDO inhibitor to improve cancer therapy. The regressions achieved by 1MT in combination therapy did not appear to reflect drug-drug interactions that raised the cytotoxicity of the chemotherapies tested, as the efficacy was increased without increasing the known side-effects of the chemotherapies tested (18). Further, T cell depletion in subjects nullified the therapeutic benefits of 1MT administration, establishing that its action was based in provoking T cell attacks in the presence of chemotherapy (18). Overall, these observations challenged the paradigm at the time that active immunotherapy and chemotherapy are fundamentally incompatible by offering one of the first demonstrations of a productive immunochemotherapy regimen based exclusively on small molecule drugs (41).

Careful biochemical studies with purified IDO1 enzyme revealed that only the L racemer of 1MT exerted any catalytic inhibitory activity (42), and it became apparent that L-1MT is actually a weak substrate rather than a true catalytic inhibitor of IDO1 as discussed in detail elsewhere (43). Unexpectedly, the D racemer lacking enzyme inhibitory activity was actually more potent in empowering chemotherapy as well as relieving T cell suppression by IDO1-positive dendritic cells from mouse or human sources (42), although there are conflicting data on T cell suppression (44, 45). Mouse genetic studies were consistent with IDO1 pathway targeting in showing that the anticancer efficacy of D-1MT relied genetically on the presence of a functionally intact IDO1 gene (42), similar to *bona fide* IDO1 enzyme inhibitors (24, 25). However, subsequent studies of D-1MT make it clear that its antitumor effects in cells and in animals is likely to be complex (7, 43). Indeed, mechanistic studies have made it clear that neither racemer of 1MT is a valid probe of IDO1 enzyme activity, a question ultimately addressed by isolation of several unique structural classes of true enzymatic inhibitors with related antitumor properties, as reviewed elsewhere (7). Cellular mechanisms of action for indoximod have been defined which involve relief of suppression of Teff cells in tumors, limitations on the generation of Tregs, and re-programming of Tregs to Th17 helper cells in draining lymph nodes (Figure 1) (2, 46, 47). The robust preclinical efficacy of D-1MT/indoximod in combination with DNA damaging chemotherapy led to its inclusion on a list of ‘top ten' agents for clinical evaluation by an NCI immunotherapy workshop (48, 49). In 2008, a decision was made to advance D-1MT/indoximod (NLG-8189) to first-in-man trials as a single molecular species through an FDA investigational new drug application by a collaborative team of investigators from the Medical College of Georgia, Lankenau Institute for Medical Research, National Cancer Institute and NewLink Genetics Corporation as corporate sponsor.

## Clinical evaluation of indoximod

Phase 1 studies of indoximod as a monotherapy or in combination with chemotherapy showed it to be well-tolerated in patients with advanced breast cancers or other solid tumors (50, 51). In a first-in-man dose escalation study conducted in advanced breast cancer patients receiving standard of care taxane therapy, the administration of indoximod was well-tolerated to a maximum delivered dose of 1,200 mg twice daily. Four partial responses were observed in the patients studied (*n* = 22) in the absence of any apparent drug-drug interactions (50). In a larger dose escalation study of advanced cancer patients with various solid tumors, the maximum tolerated dose was not reached until 2,000 mg twice daily (51). Notably, several patients on the indoximod trial who had been treated previously with ipilimumab developed hypophysitis, an autoimmune reaction to the pituitary gland which had been documented in patients treated with ipilimumab. In these patients, stable disease >6 months was observed, encouraging the notion that indoximod can reactivate latent T cell immunity in cancer patients. In the initial trials of indoximod, its relative apparent safety is notable given comparisons to the acute side-effects of immune checkpoint therapy, however, a case of Parkinsonism was reported recently in a patient receiving indoximod treatment (52). While safety studies were not able to identify a maximum tolerated dose (MTD) for indoximod, pharmacokinetic analysis indicated that 1,200 mg twice daily (BID) was the maximum exposure that could be achieved in a patient based on a plateau that occurred in plasma AUC and Cmax beyond this dose. Oral dosing generated a Cmax at 2.9 h with a serum halflife of 10.5 h. Interestingly, there was evidence in indoximod-treated patients of increased levels of both C reactive protein (CRP) and autoantibodies to tumor antigens, consistent with an increased inflammatory response to the chemotherapy onboard (51). Based on these initial studies, multiple Phase 2 studies of indoximod in continuous oral cycles have been conducted at a dose of 1,200 mg twice daily.

Phase 2 data from several trials of indoximod in different types of cancer has been provocative but not uniformly positive in all disease settings examined so far (Table 1). All trials have been conducted in combination with standard of care treatments, including in metastatic cutaneous, mucosal, or uveal melanoma with immune checkpoint therapy; advanced breast cancer (BRCA), acute myeloid leukemia (AML), and pancreatic ductal adenocarcinoma (PDAC) with chemotherapy; and advanced prostate carcinoma (PC) with sipuleucel-T (Provenge®), an approved dendritic cell vaccine. In particular, the melanoma and prostate trials have illustrated significant therapeutic activity of indoximod in empowering anti-PD1 treatment (pembrolizumab) and sipuleucel-T vaccine treatment (Provenge® autologous dendritic cells), respectively.

**Table 1:** Overview of indoximod clinical data (phase 1b/2, phase 2 trials).

**Disease**	**Design**	**Combination**	**Number of Patients Evaluated**	**Evidence of Efficacy?**	**NCT Reference Number**	**References**
Melanoma *cutaneous, mucosal, uveal*	Phase 2, single arm, 1200 mg bid	SOC Pembrolizumab (evaluated), Nivolimumab or Ipilimumab (non-evaluated)	85	Yes ORR 53% (PD-L1+ 77%, PD-L1– 37%) CR 18%	03301636	(53)
Prostate *metastatic CRPC*	Phase 2, dual arm, randomized 1200 mg bid or placebo	SOC sipuleucel-T vaccine	46 (24 placebo, 22 indoximod)	Yes 10.3 mos treatment vs 4.1 mos placebo (p = 0.011)	0156092	(54)
Acute Myeloid Leukemia	Phase 1b/2 dual arm (var. doses ± placebo in Phase 2)	SOC Induction + Maintenance Chemotherapy	6	High occurrence of MRD after one cycle of induction therapy in 5/6 patients	02835729	(55)
Brain *Adult glioma*	Phase 1b/2a single arm (var. doses)	SOC Temozolomide + Bevacizumab + Radiotherapy	12	SD (5-10 mos) 3/12 patients previously refractory to SOC; near PR, 1/12 pts with progressive ongoing reduction in tumor size	02052648	(56)
Brain *Pediatric*	Phase 1b/2 single arm (var. doses)	SOC Temozolomide ± Radiotherapy	29 (12 chemo, 17 chemo+radio)	Yes TTRF = 12 mos vs. 3.2 mos chemo+radio vs. chemo-only	02502708	(57)
Pancreas *PDAC*	Phase 2 single arm 1200 bid	SOC Gemcitabine or Nab-Paclitaxel	104	Some, but did not meet pre-specifed goal 30% reduction in HR. Median OS = 10.9 ORR = 46.2%	02077881	(58)
Breast *Stage IV (naïve)*	Phase 2 single arm 1200 bid	SOC Taxotere	169	No No difference in ORR, PFS, or OS	01191216	NA

*SOC, standard of care; SD, stable disease; PR, partial response; CR, complete response; HR, hazard ratio; MRD, minimal residual disease; PFS, progression-free survival; TTRF, time to regimen failure*.

### Metastatic melanoma

The initial phase 1b study in melanoma illustrated the safety of indoximod in combination with the anti-CTLA4 antibody ipilimumab, the standard of care treatment for metastatic melanoma at the time of testing. Nine patients with unresectable stage 3 or 4 melanoma patients were treated with escalating doses of indoximod (600 mg BID, then 1,200 mg BID). Unlike an IDO1 enzyme inhibitor (epacadostat) which yields dose-limiting toxicity (DLT) in combination with ipilimumab, no DLT was encountered with indoximod. Thus, the pre-specified highest dose of indoximod (1,200 mg BID) was deemed tolerable and used as the recommended phase 2 dose (RP2D) in combination with checkpoint inhibitors (59).

The phase 2 melanoma study enrolled over 100 patients in a single-arm trial of indoximod plus provider choice of immune checkpoint antibodies (ipilimumab or the anti-PD1 antibodies nivolimumab or pembrolizumab) (NCT03301636). A preclinical treatment rationale was provided by a study showing that indoximod could improve the response of B16 murine melanoma tumors to immune checkpoint therapy (60). In this single-arm trial (53), 85 patients were treated with pembrolizumab plus indoximod with on-treatment imaging to meet a pre-specified definition of evaluable for efficacy. Overall response rate (ORR) was 53% with a rate of complete response (CR) of 18% and disease control rate (DCR) of 73%. Median progression-free survival (PFS) was 12.4 months (95% confidence interval: 7.1, 24.9). Notably, these efficacy data paralleled those achieved by the approved combination of nivolumab and ipilimumab, but without the elevated rate of severe autoimmune side-effects experienced by patients treated with these agents (61). Stratifying the data by PD-L1 expression status, the ORR in PD-L1 positive (+) patients was 77% vs. 37% in PD-L1 negative (-) patients. Some responses seen in uveal melanomas were encouraging given its extremely aggressive nature and complete lack of response to immune checkpoint therapy (62). Overall, these data suggest the ability of indoximod to safely augment anti-PD1 antibody responses, strongly encouraging a randomized Phase 3 trial in this disease setting. These data are striking in light of the failure of epacadastat, a direct IDO1 enzyme inhibitor, to show any benefit to melanoma patients in the phase 3 ECHO-301 study when administered in combination with pembrolizumab. Given the different mechanism of action of indoximod, its independent evaluation must not be dismissed out of hand.

### Metastatic castrate-resistant prostate cancer

Further significant evidence of the efficacy of indoximod as an immunometabolic adjuvant has been documented in advanced prostate cancer. In a randomized study of metastatic castrate-resistant disease (NCT01560923), 46 patients treated with the dendritic cell vaccine sipuleucel-T (Provenge®) received placebo (*n* = 24) or indoximod (*n* = 22) with the latter cohort displaying a >2-fold increase in overall survival (OS) (54). Indoximod was administered for 10 weeks with 3 additional months in cases where an absence of radiographic or clinical progression was documented. Immune monitoring of patients was the same as performed for the IMPACT study which led to approval of sipuleucel-T (63). Indoximod was well tolerated with no significant difference in adverse events between the two study arms. Median OS had not yet been achieved at the time of report, but median radiographic PFS was 10.3 months in the treatment arm vs. 4.1 months in placebo arm (*p* = 0.011). Notably, the PFS on the placebo arm was identical to that reported in the pivotal IMPACT study for sipuleucel-T. These positive data align with recent evidence that epithelial-mesenchyme transition (EMT) drives IDO1 expression as part of this key step in metastatic progression of prostate cancer to its deadly castrate-resistant form (64). Overall, the findings of this randomized phase 2 trial with a placebo control arm strongly encourages further study of indoximod as an immunometabolic adjuvant for prostate cancer treatment.

### Acute myelogenous leukemia (AML)

In a Phase 1b trial that includes a randomized Phase 2a component to treat AML, patients with newly diagnosed disease received remission-induction chemotherapy (cytarabine plus idarubicin) plus consolidation chemotherapy (high dose cytarabine), a standard of care regimen, with the addition of indoximod or placebo as maintenance therapy (55) (NCT02835729). The dose escalation was a standard 3+3 design for the phase 1 portion aimed at gauging toxicities in combination with the chemotherapy regimen [400, 600, 1,000, 1,200 mg indoximod]. A different schedule was used in this trial, with indoximod provided every 8 h starting on day 8 of induction therapy, avoiding administration on days that patients received consolidation chemotherapy, and then stopping it 4 weeks prior to hematopoietic stem cell allo-transplanation. At the time of the report, the evidence presented indicated that indoximod did not add significant toxicity to standard of care treatment, and early response data suggested a high occurrence of minimal residual disease after one cycle of induction chemotherapy.

### Brain cancer

Phase 1b/2 single-arm trials in adult and pediatric brain cancers are being conducted in which indoximod is combined with chemotherapy or chemo-radiotherapy, with some early but intriguing efficacy data being reported. A preclinical treatment rationale was established in a robust orthotopic model of malignant brain cancer (glioblastoma), where the synergistic effects of indoximod were demonstrated in combination with temozolomide (TMZ) and radiation as a cooperative DNA damaging modality (65). In the latest report from the adult trial (NCT02052648) (56), 12 patients who had progressed on standard of care therapy with TMZ were enrolled in a traditional 3+3 dose escalation study of indoximod (600, 1,000, or 1,200 mg twice daily). No dose-limiting toxicity was encountered nor did indoximod cause a delay or reduction in TMZ dosing in any patient. The best responses documented were 1 patient with partial response per Response Assessment in Neuro-Oncology (RANO) criteria at 15 months and 4 patients with stable disease lasting between 4 and 11 months (66). A phase 2 expansion of the study is ongoing at the 1,200 mg twice daily dose in combination with TMZ, bevacizumab and ateriotactic radiation (SRS) (NCT02052648).

In the pediatric brain cancer trial (NCT02502708) (57), the first trial to evaluate indoximod both in children and in the context of radiotherapy, 17 patients from an original cohort of 29 heavily pretreated patients in the dose escalation phase 1b study who were eligible to receive further treatment were administered indoximod and radiotherapy followed by standard of care cycles of TMZ with indoximod as maintenance therapy. The other 12 patients received only indoximod and TMZ. Both treatments were well tolerated with minimal toxicity attributed to indoximod. Overall, at the time of the report, 29 patients in the dose-escalation phase of the study exhibited a median PFS of 6.2 months and median time to regimen failure (TTRF) of 11.7 months, which compares favorably with historical controls. Notably, patients receiving radiotherapy appeared to benefit significantly when indoximod was added, with a median TTRF of 12 months observed vs. 3.2 months without radiotherapy (*p* = 0.04). These data suggested a dose-sparing effect of indoximod on conventional chemo-radiotherapy, potentially extending efficacious responses. The notion that targeting the IDO pathway may improve chemo-radiotherapy is supported a recent study in lung cancer (67). Encouraged by these response data, the same regimen is now being tested in patients with diffuse intrinsic pontine glioma (DIPG), a dismal disease with no effective treatment option. Thus far, 3/6 patients enrolled are reported to have achieved good symptomatic and radiographic response.

### Pancreatic ductal adenocarcinoma (PDAC) and breast cancer (BRCA)

In contrast to the trials above, two phase 2 studies of >100 patients in pancreatic or breast cancer have shown little to no evidence of efficacy. In a single-arm study of metastatic PDAC (NCT02077881), 104 of 135 patients enrolled to receive a standard of care regimen of gemcidabine or nab-paclitaxel plus indoximod were judged evaluable for efficacy by a pre-specified definition (58). Patients were enrolled with treatment-naïve disease or first line therapy following earlier resection and adjuvant therapy. Treatment was administered until disease progression or toxicity occurred. Median OS was 10.9 months with an ORR of 46.2%. Notably, responding patients exhibited an increased density of intratumoral CD8+ T cells. This study did not meet its pre-specified goal of a hazard ratio (HR) = 0.70, but the increased ORR that was observed correlated with a positive immunological response. In contrast, a study of metastatic BRCA patients failed to produce any evidence of efficacy. In this study of 169 newly diagnosed patients treated with taxotere and indoximod (NCT01191216), no statistically significant difference in PFS, OS, or ORR was observed. While these two types of aggressive cancer set a high bar for improvements in efficacy, the selection of subjects who were not heavily pre-treated opened a window of opportunity for indoximod. Taken together, clinical findings clearly encourage further study of indoximod as an immunometabolic adjuvant for immunotherapy in treatment of melanoma and prostate cancer, and possibly for DNA damaging modalities in treatment brain cancer and AML, a diverse set of diseases and combinations that illustrate the potentially broad uses indoximod may realize in the clinical setting.

## Mechanisms of action of indoximod

### Relieving suppression of mTORC1 activity in T cells due to tryptophan starvation

**Figure 2:**
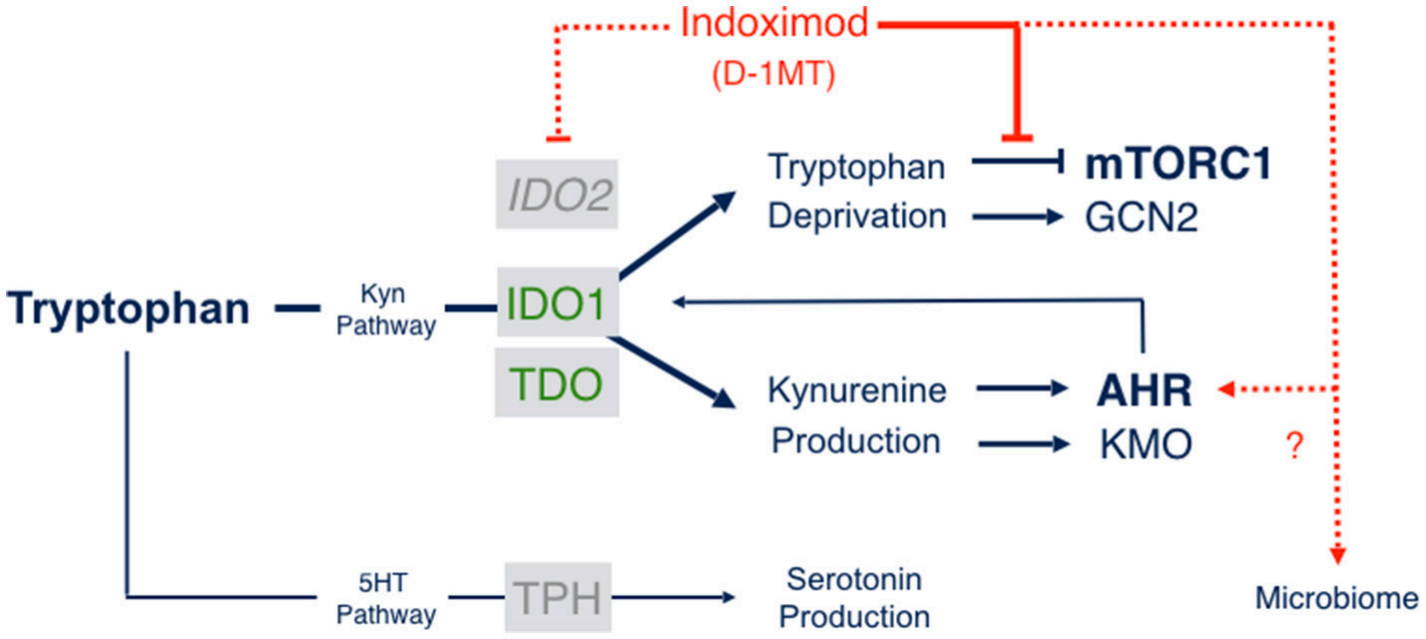
Indoximod mechanisms of action. Tryptophan catabolism proceeds through pathways leading to serotonin or NAD production, the latter through the kynurenine pathway which handles ~95% of Trp catabolism in mammals. Indoximod is a Trp mimetic which mTORC1 interprets as L-Trp under conditions of high Trp catabolism and autophagy. Thus, the drug acts to block the suppressive signal on mTORC1 function generated by IDO/TDO activity. Other suggested mechanisms of action include an indirect suppression of IDO2 activity; a modulation of kynurenine-regulated AHR function, which may also influence feedback on IDO1 expression and activity; and an influence on gut microbial physiology influencing systemic immunity (see the text).

The molecular mechanisms of action of indoximod as an inhibitor of the IDO pathway are a subject of continued study. However, only one mechanism of action has been described that is consistent with pharmacokinetic analyses of the blood serum levels of indoximod that are actually achieved in human subjects (68). Specifically, in cells subjected to IDO/TDO-mediated tryptophan depletion, indoximod has been shown to relieve suppression of the master metabolic kinase mTORC1 that occurs in tryptophan-depleted cells, with an IC50 (~70 nM) that is more potent than L-tryptophan itself (68). mTORC1 controls protein synthesis, coordinating nutrient levels to different cellular physiological responses of autophagy vs. growth. In T cells, mTORC1 is pivotal in determining autophagy/tolerance vs. growth/activation. mTORC1 is downregulated by depletion of essential amino acids like tryptophan, to which it responds by activating autophagy as an attempt to access tryptophan from intracellular stores. Accordingly, depletion of tryptophan by IDO/TDO activation downregulates mTORC1 and promotes autophagy which indoximod reverses as a tryptophan mimetic (Figure 2). Although the precise connections between IDO/TDO-mediated downregulation of mTORC1 in T cells are not well understood, there is evidence of an intermediate role for the amino acid sensing kinase GLK1 which acts upstream to regulate not only mTORC1 but also PKC-θ, a T cell receptor regulatory kinase (69). Thus, GLK1 may be a linchpin between tryptophan catabolism by IDO/TDO enzymes and mTORC1 downregulation in T cells (7).

By restoring mTORC1 activity, indoximod acts to reverse mTORC1-activated autophagy triggered by tryptophan depletion (68). Since indoximod is a D-tryptophan analog, it cannot support protein translation, but nevertheless it is interpreted by the mTORC1 kinase as a high-potency L-tryptophan mimetic. Why this is the case is unclear, but a mammalian capability to recognize (if not use) D-amino acids might reflect immune crosstalk with the microbiome given their use in bacteria (70). In any case, mTORC1 has a critical role in human Teff cell activity and indoximod acts directly in human T cells where it exerts a direct effect, unlike IDO1 enzyme inhibitors (71).

There are at least three implications of this mechanism of action. First, by targeting a downstream effector molecule, indoximod differs from IDO1 enzyme inhibitors in being agnostic to the IDO/TDO enzyme(s) contributing to cancer pathogenesis. Thus, indoximod is rationalized to treat tumor cells overexpressing IDO1, IDO2 or TDO (or any combination thereof), which is not the case for an enzyme-selective inhibitor. This is a useful feature in heterogenous plastic tumors which represent the norm in advanced cancer patients. Second, by targeting a convergent effector mechanism used by all IDO/TDO enzymes, indoximod may prove less sensitive to inherent or acquired resistance that may arise in patients due to IDO1 mutation, IDO1 overexpression or other target bypass mechanisms that heterogeneous cancers evolve. On this point, preclinical genetic studies illustrate clearly how tumoral bypass of an IDO1-specific blockade is associated with IDO1-independent elevation of regional kynurenine levels (21), suggesting the availability of resistance pathways via TDO2 or IDO2 activation. The critical question of inherent and acquired resistance to IDO1 selective blockade is discussed in greater depth in a separate review of the failed ECHO-301 phase 3 clinical trial in melanoma patients of pembrolizumab with epacadastat, a direct IDO1 enzyme inhibitor that added no benefit to the immune checkpoint therapy under the conditions of study (72). Lastly, mTORC1 is implicated in tumor cell growth and proliferation as well as in T cell activation. Thus, if indoximod also provokes mTORC1 activation in tumor cells, the drug may also empower tumor cell killing when combined with chemotherapeutic drugs, which generally exhibit greater cytotoxicity against growing cells.

Overall, the evidence that indoximod may broaden the efficacy of pembrolizumab (53) suggests that restoring mTORC1 in effector T cells might be sufficient to improve therapeutic responses with reduced risks of resistance due to IDO1 bypass. On this point, it is known that mTORC1 drives expression of ICOS, a positive-acting T cell co-regulatory receptor, and that elevated expression of ICOS in melanoma patients receiving immune checkpoint therapy correlates with the most favorable outcomes (73). In efforts to further leverage its features as an IDO/TDO effector pathway inhibitor, novel salts of indoximod and a pro-drug form of the drug (NLG-802) with superior pharmacokinetic properties have recently been described which have entered clinical testing (71).

### Other mechanisms of action

Indoximod clearly has complex immunomodulatory properties, as illustrated, for example, by its ability to act on B cells to relieve inflammation in a murine model of autoimmune rheumatoid arthritis (74, 75). Thus, other mechanisms of action that have been described for indoximod are likely to illuminate its therapeutic properties.

#### Indirect blockade of IDO2 which is implicated in IDO1-mediated treg activation

The catalytic activity of IDO2 has been shown to be inhibited indirectly by indoximod in human kidney cells where the IDO2 gene is expressed normally (76). There is conflicting data in dendritic cells, which express IDO2 as well as IDO1, on the ability of indoximod in this setting to block T cell suppression (42, 77, 78). However, mouse genetic studies support a link between indoximod action and IDO2 function, for example, in demonstrating that the therapeutic benefits of indoximod administration in a model of rheumatoid arthritis that relies on the presence of the *Ido2* gene (74), which interacts genetically with IDO1 in IDO1-mediated activation of Treg cells in the mouse (28). Here we note that the ability of indoximod to limit rheumatoid arthritis is highly relevant to combination treatments with immune checkpoint antibodies, which often cause autoimmune side-effect in patients. In this sense, indoximod co-administration with immune checkpoint antibodies may widen the therapeutic window at both ends, by extending efficacy and reducing side-effects, unlike IDO1-selective enzyme inhibitors.

#### AHR modulation

At high concentrations in cell culture (1 mM), evidence has been presented that D-1MT/indoximod can elevate transcription of IDO1 leading to increased production of kynurenine in cancer cells (79), but the concentrations used in this study, which exceed by ~100-fold the serum levels of indoximod achieved in patients in clinical trials (50), cast doubt on the physiological relevance of this observation. However, a very recent report offers additional support for the related idea that indoximod may somehow affect IDO1 expression in cell-specific ways via AHR (80), a transcription factor that binds and is activated by kynurenine (81) as a convergent effector pathway downstream of all IDO/TDO enzymes (7). Indeed, other evidence has been presented for an autocrine feedback pathway involving IDO1, AHR, and IL-6 that controls IDO1 expression in cancer cells (82).

The AHR connection for indoximod is complex. There are binding sites for AHR in the IDO1 gene and other genes that influence the differentiation of dendritic cells, T helper cells and Tregs and the proliferation of Teffs and Tregs where AHR has influence (83). In a recent study reported at the 2018 AACR conference (80), indoximod was reported to modulate AHR-dependent transcriptional activity in human liver and primary T cells, in the latter case altering the transcription of genes associated with T helper and Treg phenotypes. These effects were reversed by an AHR inhibitor, suggesting that indoximod acts upstream of AHR (80). In plasmacytoid dendritic cells *in vitro* and *in vivo* (in tumor-draining lymph nodes), indoximod was found to downregulate IDO1 expression and function, decrease kynurenine production and increase T cell proliferation, while promoting a phenotypic shift in T cells from Treg to Th17-producing T helper cells (80). Thus, in addition to resuscitating Teff cells in tumors, indoximod may also act in draining lymph nodes to reprogram the AHR effector pathway to shift Tregs to Th17 cells.

#### Perspectives of indoximod on IDO/TDO/AHR signaling to the gut microbiome

Immune homeostasis involves a dynamic balance between tolerance of commensals and suitable immune responses to eradicate or otherwise control pathogens (84, 85). Tolerance is important to avoid tissue injury but at the potential costs of chronic infections and inflammation which in the long term become factors in metabolic diseases, autoimmunity, and, in certain settings, cancer (85). Regarding indoximod mechanisms this is an important area to survey given evidence that the therapeutic impact of anti-PD1 therapy is determined by microbiome character, in both preclinical models (86, 87) and clinical settings (88–90).

Cross-regulatory circuitry between IDO1 and AHR is a key factor in mediating disease tolerance (91). For example, exposure to bacterial lipopolysaccharide will program a state of refractoriness to further LPS challenge (endotoxin tolerance), a phenomenon reflecting the engagment of AHR in long-term control of systemic inflammation only when IDO1 is active, which responds late upon initial stimulation but earlier upon subsequent challenge. Mechanistic studies have revealed a feedback control cycle, with SRC kinase as an intermediate between kynurenine-activated AHR and IDO1 expression in regulating tolerance to bacterial endotoxins, a state that protects against immunopathology in Gram-negative and Gram-positive infections. In this fundamental way, IDO1 and especially AHR contribute to immunologic host fitness (91).

IDO1 and AHR are highly expressed in the small and large intestine (92). IDO1 expression increases further during aging, a key factor in the likelihood of a positive therapeutic response to anti-PD1 treatment (93). In the intestine of adult germ-free mice, IDO1 levels are reduced suggesting that commensal microorganisms mediate the age-dependent increase in IDO1. Supporting the likelihood that it modulates mucosal immunity to intestinal microbiota, IDO1-deficient mice exhibit resistance to enteric pathogens, for example, to *Citrobacter rodentium* (94). Tryptophan catabolites produced by microbiota such as gut *Lactobacillus* can also act as AHR ligands, confounding a clear interpretation of the link between IDO1 and cancer that may involve microbiota-mediated tryptophan catabolism (85).

In melanoma studies of anti-PD-1/PD-L1 it appears that gut commensals of *Bifidobacteria* can enhance therapeutic efficacy (86, 88–90). Given evidence that indoximod can heighten the benefit of anti-PD1 therapy, it will be important to evaluate *Bifidobacteria* as a potential mediator in this effects, which raises the possibility of conceptualizing indoximod as a prebiotic substance. In one clue that this may be the case, indoximod was able to reverse the effects of IDO1 activity in models of colitis that are quieted by *Bifidobacteria* (95). While still in their infancy, studies of the effects on indoximod and the IDO/TDO/AHR pathways on gut microbial physiology and cancer immunity is a rich area for exploration.

## Potential benefits of indoximod treatment to quality of life in cancer patients

Recent studies suggest that indoximod may exert a variety of benefits as an immunometabolic adjuvant on the quality of life of cancer patients and survivors. The conditions that are improved are not critical to overall survival, but are of major importance to affected individuals and their oncologists and caregivers. As noted above, one interesting feature of indoximod is its ability to limit autoimmune arthritis in preclinical models, possibly by limiting IDO2 function implicated in this condition (96). Autoimmune joint inflammation is a common short and long term side effect of immune checkpoint therapy in cancer patients which indoximod may limit. This potential may be confirmed through long-term follow up of melanoma patients receiving combinations of indoximod and pembrolizumab in the phase 2 trial discussed above. Other beneficial effects of indoximod that have been described are behavioral, as evaluated in preclinical models of depression, anhedonia, anxiety or pain (97–100), one or more of which occur commonly in cancer patients and survivors. Given its relative safety in trials to date, it may be possible to consider uses in these settings, not only during cancer therapy but as a palliative adjunctive therapy. In summary, indoximod is a unique immunometabolic adjuvant with a wide potential range of uses to improve cancer therapy in adults and children, not only safely but with possible collateral benefits to quality of life.

## Author contributions

All authors contributed to composing the text. GP composed the figures. YZ, PG, AM, and GP made final edits to the text and figures.

### Conflict of interest statement

GP and AM disclose equity ownership in NewLink Genetics reflecting inventorship of licensed IDO intellectual property including indoximod and its uses in cancer treatment from the Lankenau Institute of Medical Research, as described in U.S. Patents Nos. 7705022, 7714139, 8008281, 8058416, 8383613, 8389568, 8436151, 8476454, and 8586636. GP additionally discloses equity ownership in Incyte and Merck; former and present advisory board roles for NewLink Genetics and Kyn Therapeutics, respectively; and a board director role for Meditope Biosciences. AM additionally discloses roles as an advisory board member and grant recipient for I-O Biotech AG, which is developing IDO vaccines for cancer treatment. YZ discloses research and travel support from NewLink Genetics and advisory board roles for Amgen, Roche Diagnostics, Novartis, Eisai, Castle Bioscience and Exelixis. The remaining authors declare that the research was conducted in the absence of any commercial or financial relationships that could be construed as a potential conflict of interest.
